# Agricultural Management Drive Bacterial Community Assembly in Different Compartments of Soybean Soil-Plant Continuum

**DOI:** 10.3389/fmicb.2022.868307

**Published:** 2022-05-04

**Authors:** Shi Chen, Lulu Wang, Jiamin Gao, Yiwen Zhao, Yang Wang, Jiejun Qi, Ziheng Peng, Beibei Chen, Haibo Pan, Zhifeng Wang, Hang Gao, Shuo Jiao, Gehong Wei

**Affiliations:** ^1^State Key Laboratory of Crop Stress Biology in Arid Areas, Shaanxi Key Laboratory of Agricultural and Environmental Microbiology, College of Life Sciences, Northwest A&F University, Yangling, China; ^2^Suzhou Academy of Agricultural Sciences, Suzhou, China

**Keywords:** agricultural management, bacterial community, soil-plant continuum, community assembly, co-occurrence networks

## Abstract

Flowering stage of soybean is an important agronomic trait, which is important for soybean yield, quality and adaptability, and is the external expression of integrating external environmental factors and endogenous signals of the plant itself. Cropping system can change soil properties and fertility, which in turn determine plant growth and yield. The microbial community is the key regulator of plant health and production performance. Currently, there is limited understanding of the effects of cropping systems on microbial community composition, ecological processes controlling community assembly in different soil-plant continuum compartments of soybean. Here, we hope to clarify the structure and assembly process of different soybean compartments bacterial community at flowering stage through our work. The results showed that intercropping decreased the species diversity of rhizosphere and phyllosphere, and phylloaphere microbes mainly came from rhizosphere. FAPROTAX function prediction showed that indicator species sensitive to intercropping and crop rotation were involved in nitrogen/phosphorus cycle and degradation process, respectively. In addition, compared to the continuous cropping, intercropping increased the stochastic assembly processes of bacterial communities in plant-associated compartments, while crop rotation increased the complexity and stability of the rhizosphere network and the deterministic assembly process. Our study highlights the importance of intercropping and crop rotation, as well as rhizosphere and phyllosphere compartments for future crop management and sustainable agricultural regulation of crop microbial communities.

## Introduction

The agroecosystem is a complex self-organizing systems with several different levels, including plants, microbes, and soils, which interact with each other and respond to different management practices (Ichihashi et al., 2020). Harnessing beneficial microorganisms is a sustainable way to optimize crop production management (Li Y. et al., 2021). Different agricultural management practices such as reduced tillage can maintain plant coverage and improve soil organic matter, increase the abundance of bacteria and fungi in soil, thereby further determining the field mechanisms of plant phenotypic microbial expansion (de Vries et al., 2020). Conservation tillage can increase the relative abundance of rhizosphere groups and cause specific responses of symbiotic network members of diazotrophic communities (Li Y. et al., 2021). The addition of green manure activates minor soil-borne taxa and increases soil microbial biomass and microbial diversity (Semenov et al., 2021). Due to the complementarity of resource demand among plants, intercropping can be utilized to better partition resources, which is conducive to improved yield and land use efficiency (Zhang et al., 2017). However, the interaction of microorganisms, nutrients and enzymes in the intercropping system will lead to the increase or decrease of microbial biomass and diversity. For example, Chinese milk vetch-rape intercropping reduces microbial biomass such as bacteria/fungi in rhizosphere soil of rape (Zhou et al., 2019), and proso millet-mung bean intercropping increases bacterial α diversity, thereby improving soil nitrogen availability and plant nitrogen accumulation (Dang et al., 2020). Crop rotation can ameliorate soil quality, crop yield and nitrogen use efficiency, and ensure the balance between economic and environmental benefits. Combined fertilization can also greatly improve soil versatility, and establish soil physical and chemical characteristics that affect microbial community composition, structure and nutrient cycling function, thereby exerting profound influence on the soil microbial ecosystem (Cui et al., 2013; Murtaza et al., 2017; Wang et al., 2017; Ai et al., 2018; Schmidt et al., 2019; Gao et al., 2020; Li M. et al., 2021). These agricultural cropping systems are designed to maintain environmental sustainability and agro-ecosystem services, and the microbial communities present exert an important effect in agro-ecosystems (Delgado-Baquerizo et al., 2016; Banerjee et al., 2019).

Microorganisms are one of the key components of the soil ecosystem, and can affect global ecosystem services through known and as yet to be discovered pathways (Fierer, 2017). This is especially true in agricultural ecosystems, where soil microbial communities regulate many processes such as carbon and nitrogen cycling, organic matter decomposition, and plant immunity and disease resistance (van der Heijden, 2010; Ma et al., 2018; Crowther et al., 2019; Jiang et al., 2020; Fan et al., 2021). Plants live in biogeochemically diverse soils, and soil habitat is an extremely rich microbial pool selected by host microorganisms. There are different bacterial communities present on and within the various organs and tissues of plants. A previous systematic assessment of community members revealed the many characteristics of bacteria adapted to these habitats by comparing the varied compartments of plant rhizosphere, root, and leaf (Bulgarelli et al., 2013). At the same time, integrating beneficial plant microbial groups into agricultural production can help people to improve food production safety, resource utilization and environmental protection (Busby et al., 2017). Co-evolution theory shows that plants attract beneficial microorganisms by releasing signal molecules, and then exert a selective pressure through use of the immune system and by providing specific nutrients and habitat types (Hacquard et al., 2015; Foster et al., 2017; Cordovez et al., 2019; Xiong et al., 2021b). The chemicals secreted through plant roots into the soil, alter soil properties, and regulate their growth environment. The interaction between plants and soil determines the coexistence, succession and productivity of plants in natural and agricultural systems (Hu et al., 2018).

Microbial assembly in soil is affected by different factors such as host environmental factors (Xiong et al., 2021a). The assembly of plant microbial groups begins soon after sowing and changes throughout the plant growth cycle under the influence of deterministic (such as selection mediated by biological and abiotic factors) and stochastic (such as diffusion and drift events) processes (Bulgarelli et al., 2013; Müller et al., 2016; Hassani et al., 2018; Ichihashi et al., 2020; Xiong et al., 2021a). The assembly and stability of plant microbial groups are also strongly affected by microbe-microbe interactions. The potential microbial interactions in different habitats can be analyzed through the use of microbial symbiotic networks (Agler et al., 2016; van der Heijden and Hartmann, 2016; Duran et al., 2018; Toju et al., 2018). However, there are few reports on the assembly and interaction of microbial communities in the soybean soil-plant continuum of compartments under different tillage practices.

Here we explored the impact of three agricultural cropping systems (CC,CI,CR) on bacterial community structure in different compartments of soybean soil-plant continuum. We aimed to address the following questions: (a) Do different agricultural cropping systems affect the composition and structure of the soil-plant bacterial community within the various compartments of soybean? (b) Is there any difference in the complexity and stability of the networks among continuous cropping, intercropping, and crop rotation systems? (c) What are the assembly processes of the bacterial communities from different soil-plant continuum compartments under the three cropping systems?

## Materials and Methods

### Field Trial and Treatments

Field experiments were conducted in Suzhou (33° 63′ 66.6″ N, 117° 08′ 85.7″ E, altitude 30 m), Anhui Province (East China). The soil type is the Fluvisol according to the FAO soil classification system (Micheli et al., 2006), annual precipitation is 857 mm and annual mean temperature is 14.4°C. The field experiment was begun in the summer of 2019, using a split plot design (Supplementary Figure S1), and samples were taken in 2020. The primary treatment was cropping system, and the secondary treatment was nitrogen fertilization. Four plot groups were present in the experiment, and each treatment was repeated four times, with the area of each plot being 20 m^2^. 1 m protective rows were set between the four repeated primary treatment groups, and 0.5 m protective rows were set between the areas with different nitrogen fertilizer measures in each secondary treatment plot. The field experiments included three cropping systems [(1) CC: soybean continuous cropping; (2) CR: soybean-wheat crop rotation; (3) CI: soybean-maize intercropping], and five nitrogen fertilizer treatments [(1) CK: plots with no nitrogen fertilizer; (2) IN_1_: 50% inorganic nitrogen fertilizer (20 kg N/hm^2^/year); (3) IN_2_: 100% inorganic nitrogen fertilizer (40 kg N/hm^2^/year); (4) ON_1_: 50% organic nitrogen fertilizer (4,000 kg/hm^2^/year), (5) ON_2_: 100% organic nitrogen fertilizer (8,000 kg/hm^2^/year)]. In the soybean-maize intercropping system, due to the growth needs of maize, the nitrogen application rates of IN_1_, IN_2_, ON_1_, and ON_2_ treatments were 55 kg N/hm^2^/year, 110 kg N/hm^2^/year, 11,000 kg/hm^2^/year, and 22,000 kg/hm^2^/year, respectively. Phosphorus fertilizer (100 kg/hm^2^) and potassium fertilizer (100 kg/hm^2^) were used as base fertilizer in each intercropping treatment. All the organic and chemical fertilizers were added annually and fully mixed with soil before planting. Planting method involved bar sowing with row spacing of 40 cm, shallow plowing before sowing and management according to local practice. The soybean variety was semi-dwarf, strong lodging resistance and suitable for local planting Zhonghuang 13, crop rotation wheat and intercropping corn were the same as those used by local farmers and the rotation period was 1 year.

### Sample Collection

**Figure 1:**
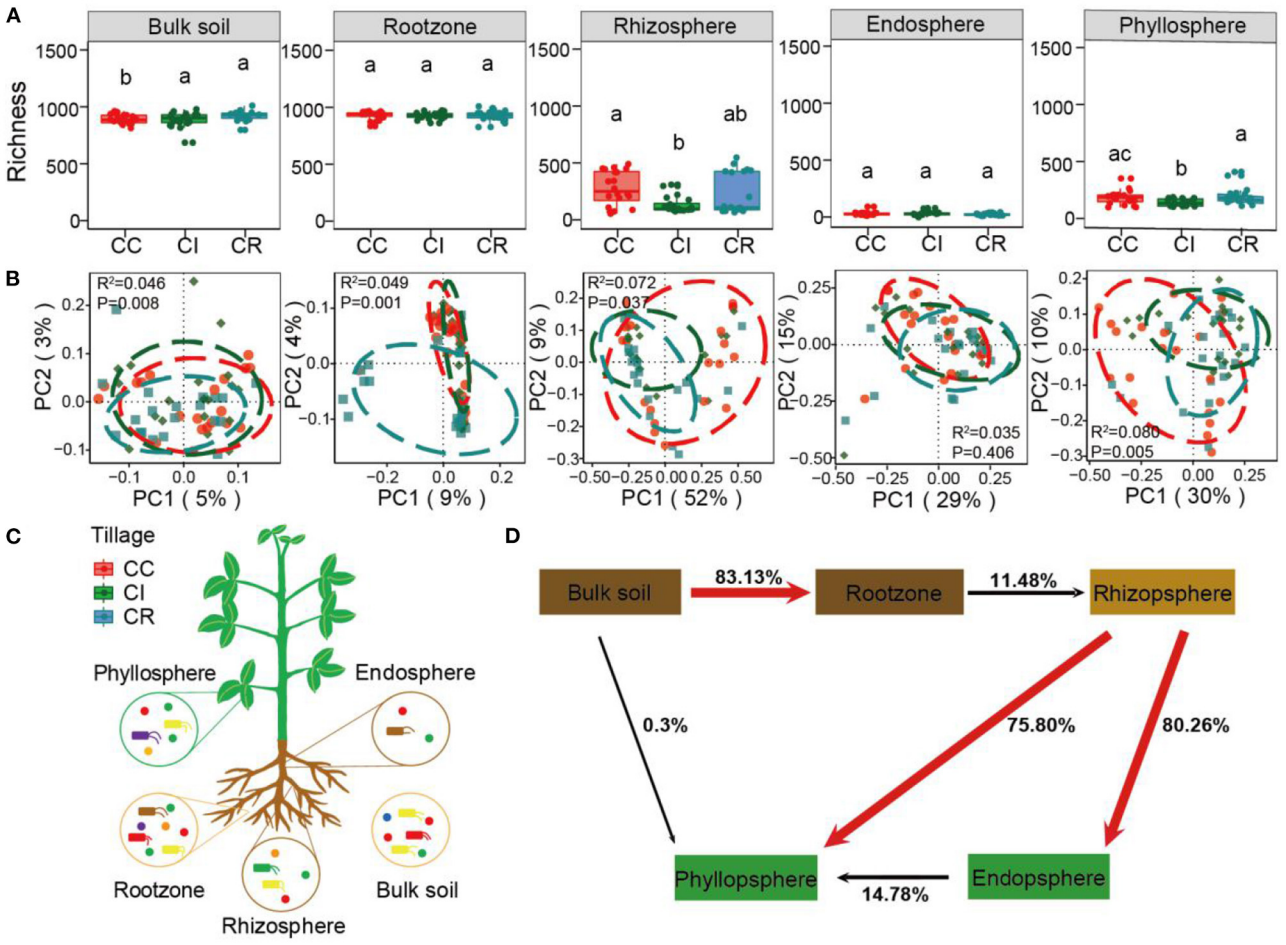
The α diversity and β diversity of bacterial communities in soil-plant continuum compartments under continuous cropping (CC), intercropping (CI), and crop rotation (CR) systems. **(A)** Represents the richness index calculated by the bacterial ASVs table. According to PCoA determination **(B)**, the bacterial community structure of three cropping systems (CC, CI, and CR) in five soil-plant continuum compartments (bulk soil, root zone, rhizosphere, endosphere, and phyllosphere). A 85% confidence ellipse with the same color as the patch was shown around the sample. **(C)** Is based on soil-plant continuous compartment niche sampling layout and **(D)** is a plant microbiome source model (SMPM) showing potential sources of soil-plant continuum-associated bacterial communities for each niche. The same lowercase letters indicate no statistically significant (*P* < 0.05) difference between cropping systems.

During the flowering stage of soybean (August 11, 2020), the five-point sampling method is adopted and the edge effect is excluded. The bulk soil was collected by auger corer (0–15 cm deep, about 20 cm away from the plants and removal of 2 cm surface plant litter), and five sub-samples were fully mixed to create composite biological samples for each plot. In the bulk soil portion, impurities such as stones were removed by sieving through a 2 mm grid. When the rootzone, rhizosphere, and leaves were sampled at the same site, 6-8 soybean plants were selected from each plot by the five-point sampling method. 1-2 leaves in the upper and middle positions of the plant were cut off, immediately placed into a bag, and held on ice. Then the tightly associated soil surrounding the root system of the plant (defined as rootzone soil) was collected by shaking. Part of the soil adhered to the root surface of 2 mm and washed away by PBS buffer and ultrasonic oscillation was defined as rhizosphere soil, and the rest was root samples (Xiao et al., 2017). All leaf, root and soil samples were stored in dry ice and transported to the laboratory. In total five samples were finally obtained: bulk soil, rootzone soil, rhizosphere soil, endosphere, and phyllosphere (Figure 1C). Then all samples were divided into two parts and stored at −80°C and 4°C, respectively, for subsequent DNA extraction and physical and chemical analysis.

### Bacterial DNA Extraction and 16S rRNA Gene Amplification

Soil microbial DNA was extracted from 0.5 g fresh soil by using the Fast DNA SPIN Kit (MP Biomedicals, Santa Ana, CA) according to the manufacturer's instructions. Extraction of plant microbial DNA from 0.5 g fresh roots and leaves was performed using the CTAB method. The bacterial 16S rRNA gene was targeted using the primer pair 515F (5′-GTGCCAGCMGCCGCGGTAA-3′)/907R (5′-CCGTCAATTCCTTTGAGTTT-3′) (Cai et al., 2016). All samples were mixed at equimolar concentrations and then paired end sequencing was performed on the Hiseq2500 platform (Illumina Inc.) (Jiao et al., 2019a).

### Bioinformatic Analysis

Paired-end sequencing data of Illumina HiSeq2500 platform were spliced by flash (Magoč and Salzberg, 2011) software with overlap reads, and using Quantitative Insights Into Microbial Ecology (QIIME) software to filter splicing data (Caporaso et al., 2010), filter out the low-quality base sequences with mass fraction (Q) <20 and chimera sequences in splicing sequences. Furthermore, DADA2 was used to process raw sequencing reads for each sample (clean data), infer the unique amplicon variant (ASV) through error-corrected reads and further quality control through error model, and also filter chimeras using DADA2 pipeline (Callahan et al., 2016). Then, based on Bayesian algorithm, we used the SILVA reference database (v.12.8) to classify representative sequences from each ASV. Subsequently, a total of 17 268 169 high-quality reads were obtained by paired-end sequencing and 113,573 ASVs were assembled. Finally, we removed rare bacteria (<20 reads) and non-bacterial ASVs (Archaea, chloroplasts and mitochondria were 219, 282, and 134 ASVs, respectively) (Xiong et al., 2021b). Overall, we identified 10 053 bacterial ASVs across all samples, and the rarefaction curve reached saturation, indicating the sequencing depth was sufficient (Supplementary Figure S2). In order to further estimate the alpha diversity, we used the rarefaction method to adjust the samples with different library sizes. After rarefied, the reads threshold of each sample was 2260.

### Statistical Analysis

Microbial diversity and community composition were analyzed at the ASV level with the phyloseq (McMurdie and Holmes, 2013) and vegan (Dixon, 2003) packages in R. The measure of diversity was assessed using the richness index and ACE abundance of bacterial samples at the ASV level. The “EasyStat” package was used to test the abundance of microbial composition at the phylum level and obtain the results of significant. Bacterial beta-diversity was evaluated by calculating the Bray-Curtis distance matrices and visualized by principal coordinate analysis (PCoA). Based on Bray-Curtis distances, using the “adonis” function of vegan packages in R, PERMANOVA was used to test the significance of different cropping systems on community heterogeneity (Oksanen et al., 2015). We used SourceTracker (v.1.0) (Metcalf et al., 2016) based on Bayesian method to estimate the source and host niche of bacterial communities in different compartments of the soil-plant continuum. To explore cropping-sensitive taxa, we used the “multipatt” function in the indicspecies package in R to determine the indicator groups of each cropping system.

We used a bipartite network to visualize the sensitive and significant (*p* < 0.05) indicator species ASV in the soil-plant continuum for different compartments under the three cropping systems. The networks were constructed using the Fruchterman-Reingold layout as implemented in the R package igraph (Hartman et al., 2018). Phylogenetic trees were annotated and visualized in the itols software (Segata et al., 2013). Prokaryotic taxonomic group (FAPROTAX) functional annotation database was used to predict the geochemical material cycle processes of the bacteria (Louca et al., 2016).

Co-occurrence networks of soil-plant continuum for different compartments under CC, CI, and CR cropping systems were constructed to understand the structure and ecological interactions of the bacterial community. Spearman correlation scores were calculated, and only robust (Spearman's r >0.7 or r < −0.7) and statistically significant (*P* < 0.01) correlations were retained. Network visualization and analysis were conducted using Gephi (Bastian et al., 2009). Each node represented one ASV and each edge represented a strong and significant correlation between two nodes. We calculated a set of metrics to describe the network topology, including the characteristic path length, network diameter, and clustering coefficient. The topological characteristics of nodes were calculated at the node level, where the degree referred to the number of edges connected to a specific node in the network, the closeness centrality determining the position of a node in the network according to the distance from a specific node to all other nodes, and the betweenness centrality indicating the potential impact of a particular node on other nodes (Jiao et al., 2016, 2019b). Then, we used the inbuilt test function of stats package in R, and compared the node attributes between different compartments under the three farming systems based on the two-sample Kolmogorov-Smirnov test (Banerjee et al., 2019). The network complexity was defined according to previous studies: higher average degree represents higher network complexity (Wagg et al., 2019). Natural connectivity of a complex network was applied to reveal the stability of a network (Shi et al., 2020). When assessing the potential contribution of neutral processes to microbial community assembly, we used neutral assembly (dominance test) models to predict the relationship between the occurrence frequency of ASVs and their relative abundance (Sloan et al., 2006; Jiao et al., 2021). The model evaluated whether the microbial assembly process of the bacterial community conformed to niche-based process (prediction outside the model) or neutral model (prediction inside the model). Furthermore, we applied the normalized stochasticity ratio (NST) to evaluate the bacterial community assembly process. NST is a quantitative index with 50% as the boundary point of deterministic dominant (< 50%) and stochastic dominant (>50%) (Sloan et al., 2006; Ning et al., 2019). We used NST to evaluate the assembly process as our research was local/landscape scale sampling and our sample size met the method requirements (*n* ≥ 6). The analysis was carried out in R using the “NST” software package.

## Results

### Diversity and Composition of Bacterial in Soil-Plant Continuum Under Different Cropping Systems

PERMANOVA analysis of the complete dataset suggested that the variation in bacterial community was mainly explained by soil-plant continuum compartment (Supplementary Table S1). Subsequently, we analyzed the effects of main factor cropping and cofactor fertilization on soil bacterial community, and found that cropping systems significantly affected the microbial community in various parts of soil-plant continuum (Supplementary Table S2). Therefore, we focus on the effects of different cropping systems (CC, CI, CR) on soil-plant continuum compartments here and in subsequent analysis.

We conducted separate bacterial community profiling of 300 soil-plant continuum samples, including 60 samples in each compartment (bulk soil, rootzone, rhizosphere, soybean endosphere, and phyllosphere). We observed that the cropping management changed bacterial α diversity (Richness and ACE) in different compartments of the soil-plant continuum (Figure 1A and Supplementary Figure S3), the richness of bulk soil, rhizosphere and phyllosphere compartment under intercropping was lower than that under crop rotation and continuous cropping, while the richness of bulk soil and phyllosphere under crop rotation was higher than that under continuous cropping, but lower in other compartments (Supplementary Tables S3, S4). Thus, the direction of effect among the three cropping systems was different. The various compartments of the soil-plant continuum present different microbial habitats, each with its own specific set of organisms. The relative abundances of *Proteobacteria, Acidobacteria*, and *Gemmatimonadetes* were higher in bulk soil and rootzone compartments, far away from the plant roots. However, in the rhizosphere, endosphere, and phyllosphere which are greatly affected by plants, *Proteobacteria* was the main microbial group, and some *Firmicutes* were observed in the endosphere and phyllosphere. In addition, the abundance of *Proteobacteri*a was the highest under intercropping management (Supplementary Figure S4). Further, we compared the abundance of the top 10 microbes at phylum levels from various compartments of the soil-plant continuum under the three cropping systems and calculated the significant among them by wilcoxon test (Supplementary Table S5). Interestingly, we found that the abundance of *Proteobacteria* under intercropping was lower than that under crop rotation in bulk soil and rootzone compartments, but the abundance of *Proteobacteria* under intercropping was significantly higher than that under rotation and continuous cropping in rhizosphere, endosphere and phyllosphere (Supplementary Figure S5).

The relative abundance of most microorganisms, such as *Acidobacteria* and *Bacteroidetes* in the rhizosphere and phyllosphere, were lower under intercropping, and there were significant differences between intercropping and continuous cropping. We also found that the relative abundance of most microorganisms in the rhizosphere and phyllosphere under intercropping was lower, including *Actinobacteria, Acidobacteria, Chloroflexi, Bacteroidetes*, and *Planctomycetes*, and there was a significant difference from continuous cropping (with *Proteobacteria* displaying the opposite trend) (Supplementary Figure S5).

Principal coordinate analysis (PCoA) analysis revealed that bacterial communities from various compartments of the soil-plant continuum (except endosphere) under different cropping systems formed distinct clusters in ordination space with significant differences observed at taxonomic levels (Figure 1B, Supplementary Table S6). Furthermore, hierarchical clustering analysis revealed a clear and separate clustering among different compartments (Supplementary Figure S6). Interestingly, the rhizosphere and phyllosphere samples were clustered together. we further applied source-tracking analysis to explore the microbial sources and found that phyllosphere bacterial communities were mainly derived from the rhizosphere soil (Figure 1D).

### Identification of Indicator Species in the Soil-Plant Continuum Under Different Cropping Systems

**Figure 2:**
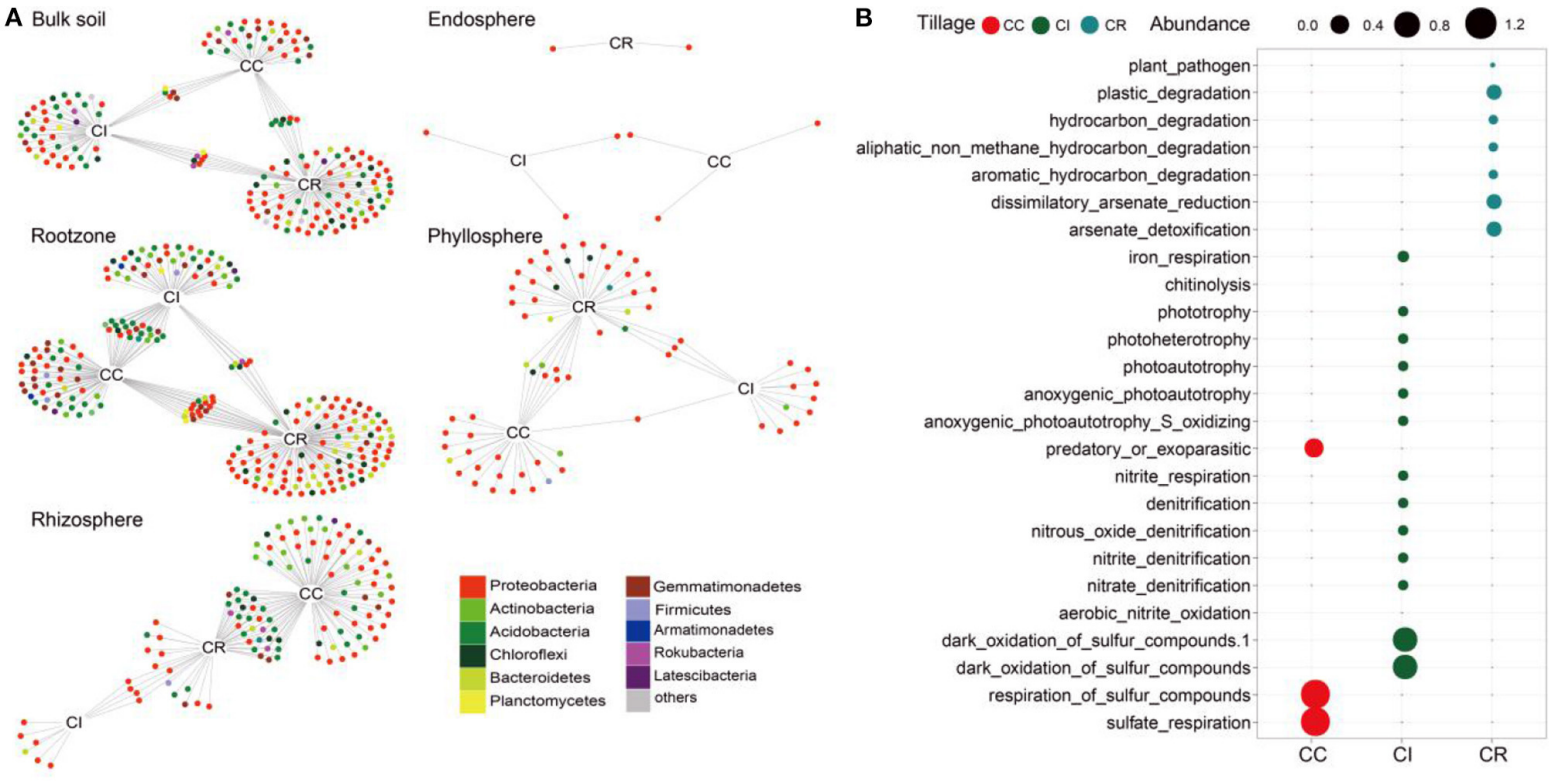
Indicator species analysis results of bacterial community in soil-plant continuous chamber under continuous cropping, intercropping and crop rotation. **(A)** Bipartite networks display cropping systems specific ASVs in the soil-plant continuum compartments bacterial communities as determined using indicator species analysis. ASVs are colored according to their Phylum assignment. **(B)** Bubble diagram showing that FARPROTAX predicted the relative abundance of ecosystem functions of ASVs sensitive to continuous cropping (CC), intercropping (CI), and crop rotation (CR) systems. Circles represent individual bacteria ASVs that are positively and significantly associated (*p* < 0.05) with one or more of the cropping systems [association(s) given by connecting lines].

Indicator species analysis was employed to identify soil-plant continuum bacteria whose abundances varied between the different cropping systems, and we used bipartite network to present the summary results (Figure 2A). We found that there were more indicator species numbers affected by crop rotation in the bulk soil, rootzone, and phyllosphere compartments, while there were more indicator species numbers affected by continuous cropping in the rhizosphere compartment. The endosphere compartment was the least affected by the three cropping systems, being only colonized by *Proteobacteria*, and there was no other microbial response to the three cropping systems. In addition, in the rhizosphere and phyllosphere compartments, the number of indicator species affected by intercropping was less than that of continuous and crop rotation, and consisted of only two microorganisms (rhizosphere was *Proteobacteria* and *Bacteroidetes* while phyllosphere was *Proteobacteria* and *Actinobacteria*). In the endosphere and phyllosphere compartments, *Actinobacteria* indicator species did not respond to intercropping and crop rotation systems (Supplementary Table S7).

As an approximation for “effect size” of cropping systems on microbial communities, we calculated the proportion of these bacterial ASVs in the total soil-plant continuum community sequence (Supplementary Table S8). In different soil-plant continuum compartments, we identified 159, 141, and 281 ASVs for continuous cropping, intercropping and crop rotation systems, corresponding to an effects size of 2.51, 10.58, and 9.91%, respectively. The effect size indicated that intercropping and crop rotation had a greater impact on soil-plant continuum bacteria community, which was 3–4 times of continuous cropping. We also constructed phylogenetic trees of ASVs specific to the three cropping patterns, and showed the abundance of these ASVs in various compartments of soil-plant continuum. It was found that the abundance of *Acidobacteria* in each cropping system was higher than that of other microorganisms, especially for the rotation system (Supplementary Figure S7). Subsequently, we predicted their functions and found that the functions of specific indicator species under different cropping systems also varied, the results showed that the microorganisms sensitive to continuous cropping were mainly involved in dark oxidation of sulfur compounds and respiration of sulfur compounds, and the abundance was relatively balanced, which the microorganisms sensitive to intercropping mainly responded to the nitrogen and sulfur cycle processes. Crop rotation was more conducive to the degradation of plants, plastics, and aromatic compounds, and metal salts (Figure 2B). In addition, we were interested in the attribution relationship among indicator species, specific species, and shared species. It was found that the number of ASVs belonging to shared species (core microorganisms) of indicator organisms sensitive to cropping patterns was greater than that belonging to species specific to a particular cropping system, which reflected the importance of these indicator species in the environment and provided some insights for us to consider the function of core microorganisms in the ecosystem (Supplementary Figure S8, Supplementary Table S9).

### Cropping System Effects on Bacterial Co-occurrence Patterns in Different Compartments of Soil-Plant Continuum

**Figure 3:**
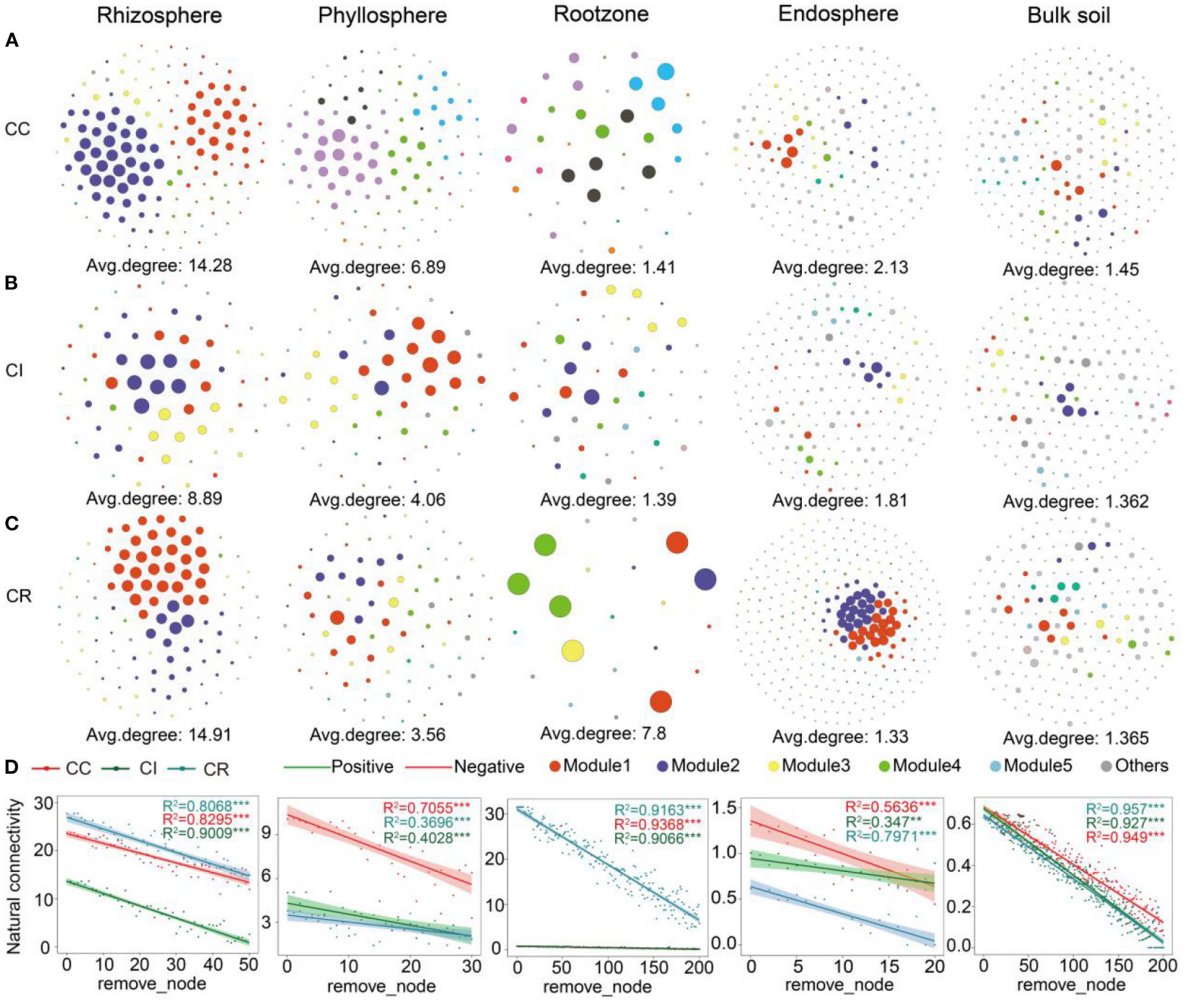
Bacterial co-occurrence networks in different soil-plant continuums compartments under **(A)** continuous cropping (CC), **(B)** intercropping (CI), and **(C)** crop rotation (CR) systems (*n* = 300). **(D)** Natural connectivity of microbial networks in different compartments of three cropping systems.

Due to the significant differences of bacterial community structure in various compartments of the soil-plant continuum among the three cropping patterns, we further evaluated the bacterial network of each cropping system. Our results suggested that the networks displayed remarkable differences in their complexity and stability (Supplementary Table S11). Kolmogorov-Smirnov test showed that node degree, betweenness centrality, and closeness centrality were significantly (*P* < 0.01) different between the three farming systems (Supplementary Table S10). The bacterial network complexity in rhizosphere was the highest, followed by that in phyllosphere, and different cropping systems had different effects on the network complexity of the various compartments (Supplementary Table S11). Crop rotation had the greatest impact on complexity (average degree) of the bacterial networks in rhizosphere (14.91) and rootzone (7.8), while continuous cropping had a greater impact on bulk soil (1.45), endosphere (2.13), and phyllosphere (6.89) (Figures 3A–C). Further, we found similar results when exploring the natural connectivity of the networks (Figure 3D). The modules were usually composed of highly interconnected microbial communities. Considering that associations in microbial networks may represent ecological interactions or niche sharing among microorganisms, we explored the interactions among microorganisms in various compartments of the soil-plant continuum networks under different cropping systems. The response of rhizosphere microbial interactions to the three cropping systems was larger than those for the other compartments. Crop rotation might have a stronger effect on interaction or shared niches in the rootzone and rhizosphere compartments, and the endosphere microbial interactions were less and its shared niche width was narrower (Supplementary Figure S9, Supplementary Table S12).

### Bacterial Community Assembly Process Under Three Cropping Systems

**Figure 4:**
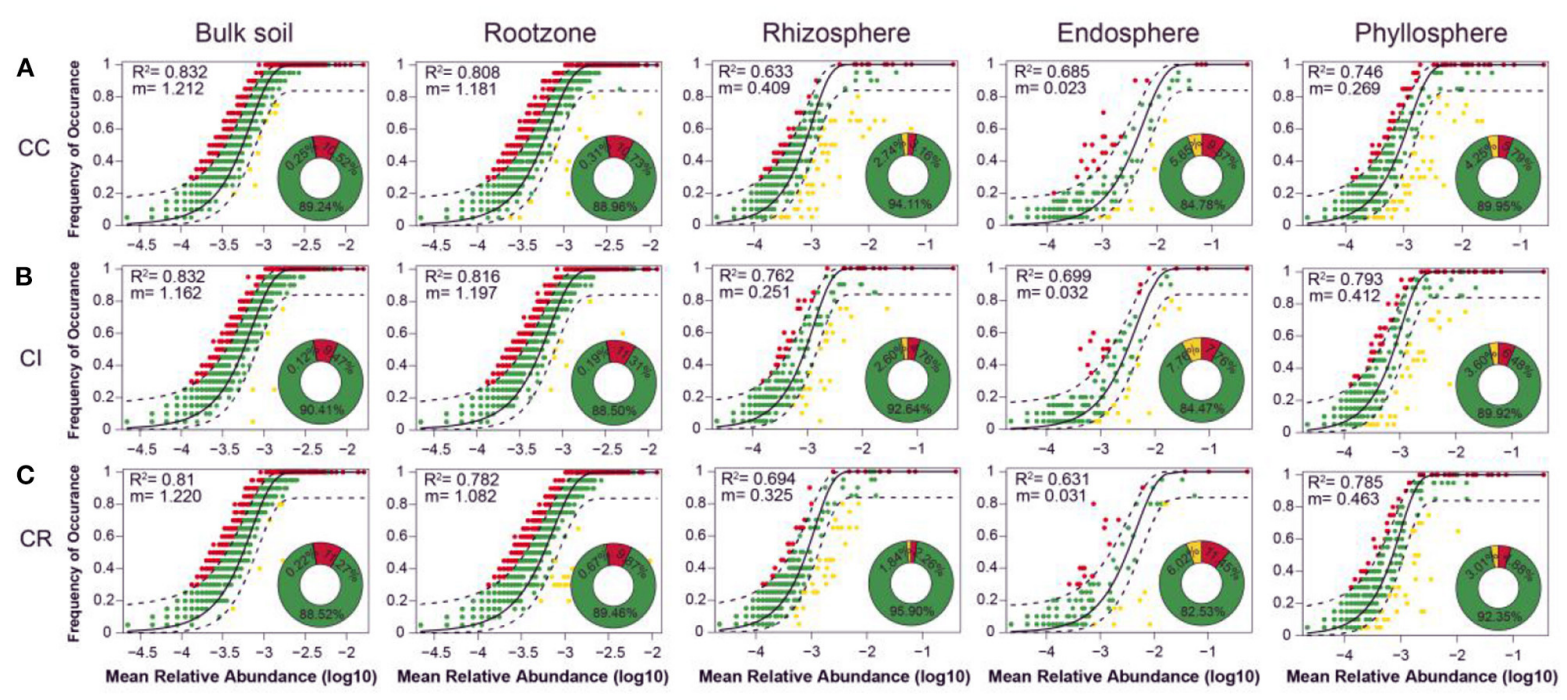
Measurement of bacteria community assembly process of different soil-plant continuum under **(A)** continuous cropping (CC), **(B)** intercropping (CI), and **(C)** crop rotation (CR) systems by dominance test: ASVs that occur more frequently than predicted by the model are shown in red, while those that occur less frequently than predicted are shown in yellow. ASVs that occur within prediction are shown in green. Dashed lines represent 95% confidence intervals around the model prediction (black line).

Based on R^2^ values and the proportions of outlying taxa beyond the dashed line reflecting those outside model predictions, the dominance test showed that bacteria community assemblages of various compartments in soil-plant continuum compartments were well-described by neutral-based models. The m value (migration rate) varied with different farming systems, reflecting the difference in the degree of limitation of species diffusion in microbial communities. The results showed that the bacterial communities of the bulk soil, rootzone, and phyllosphere had a high explanation rate (R^2^) under all cropping modes, indicating that stochastic processes were important for bacterial community assembly in various compartments of the soil-plant continuum under different cropping systems. However, the endosphere and rhizosphere communities were relatively less affected by stochastic processes. Continuous cropping affected the stochastic assembly processes of the microbial community in bulk soil, while intercropping affected the stochastic assembly processes of the communities in rootzone, rhizosphere, endosphere, and phyllosphere (Figure 4).

The NST based on Jaccard distance (NSTjac) index showed that the rhizosphere bacterial community assembly under intercropping and crop rotation was mainly controlled by deterministic processes, while the rhizosphere bacterial community assembly under continuous cropping was controlled by stochastic processes, as in the other compartments. The effect of stochastic processes on community composition in plant compartments (endosphere and phyllosphere) was slightly stronger than that in soil compartments (bulk soil and rootzone). Thus, intercropping affected the deterministic processes of the rhizosphere community and the stochastic processes of the rootzone, endosphere, and phyllosphere communities (Figure 4, Supplementary Figure S10).

## Discussion

Due to the over-cultivation and degradation of cultivated land, Fu-suo Zhang et. al proposed that intercropping could achieve sustainable intensification of agriculture and improve food production based on the current state of agriculture in China (Li C. et al., 2020; van der Werf et al., 2020). The Ministry of Agriculture of the People's Republic of China issued the “Project plan of agricultural resources and ecological environmental protection (2016–2020),” requiring crop rotation, including a fallow period, to be implemented from 2016 (Wang et al., 2019). Thus, it is essential to understand how the microbial communities in various compartments of the soil-plant continuum are affected by agricultural practices. Previous studies mainly focused on the comparison of rhizosphere and bulk soil microbial communities under long-term agricultural management, the understanding of how cropping system affects soil-plant continuum microbial communities under short-term management remains limited (Bennett et al., 2017; Teste et al., 2017; Fan et al., 2020, 2021; Xiong et al., 2021b). Here we showed that the diverse compartments of the soybean soil-plant continuum under three cropping systems (CC, CI, CR) had dissimilar bacterial communities, and there were also differences in the complexity and stability of the network. The complexity of soybean rootzone and rhizosphere networks under crop rotation was higher than that in the other cropping systems, and the assembly of the rhizosphere community in intercropping system was greatly affected by deterministic processes. Thus, crop rotation and intercropping systems can integrate the characteristics of beneficial plant microbial groups, and rotation, especially, has the potential to regulate rhizosphere microbial communities, promote plant growth through feedback regulation (Finkel et al., 2020), improve nutrient use efficiency, and improve disease resistance (Li H. et al., 2021). Therefore, in the short-term field management, we can focus more on regulating crop rhizosphere microorganisms under intercropping and crop rotation systems.

### Bacterial Community Characteristics in Different Cropping Systems

Our results revealed that bacterial community assembly in the various compartments of the soil-plant continuum was affected differently by cropping systems under short-term field farming conditions. Bulk soil and rootzone microbiota harbored a more diverse array of bacterial taxa than the rhizosphere, endosphere and phyllosphere, which was consistent with previous studies that there is a rapid loss of diversity from soil to root and aboveground tissues was due to the increased host specificity at the root-soil-crown interface (Trivedi et al., 2020; Sun et al., 2021). The α diversity of crop rotation in bulk soil was significantly higher than that of continuous cropping, but there was no significant change between the two cropping systems in plant-related compartments, indicating that crop rotation can significantly change the microbial α diversity of bulk soil. In addition, the changes of chemical composition and quality of plants for nutrient competition and input to soil in time were greater than those in space, resulting in no significant changes in microbial α diversity in a short-term field experiment of crop rotation and continuous cropping (Tiemann et al., 2015). The diversity of intercropping was the lowest within the rhizosphere and phyllosphere, which was lower than continuous cropping and significantly different from crop rotation. This may be due to the competitive niche between maize and soybean under the intercropping system. Maize secretes root exudates that improve soybean nodule formation and nitrogen fixation, while the fixed nitrogen from soybeans is transferred to maize through root exudates or mycorrhizal fungi (Li et al., 2016; Thilakarathna et al., 2016). Jessica et al. found that the soil microbial diversity of 1-year melon-cowpea intercropping system decreased, which was consistent with the results of this experiment (Cuartero et al., 2022). Studies have also shown that intercropping reduced the biomass of bacteria and fungi in the rhizosphere of rape (Hartmann et al., 2015; Lupatini et al., 2017; Sun et al., 2021). To date, no consensus has been reached on the increase, non-indigenous or decrease of α diversity caused by intercropping system (Zhang et al., 2015; Fu et al., 2019; Cuartero et al., 2022).

In addition, the relative abundance of most microorganisms in the rhizosphere and phyllosphere under intercropping was lower than that of continuous cropping and crop rotation, and there was also a significant difference between intercropping and continuous cropping. Studies have shown that these microbes may compete for nutrients in limited space, resulting in decreased microbial abundance, and rhizosphere microorganisms are closely related to phyllosphere microorganisms (Peiffer et al., 2013; Wagner et al., 2016; Hu et al., 2018). Our further traceability analysis not only showed that the bacterial communities related to crops were mainly from the bulk soil, and were gradually filtered and enriched by different niche spaces. Intriguingly, we found that the majority of microorganisms in the phyllosphere were from the rhizosphere, which might be related to the crop variety and development period of the crop. Recent studies have also shown that most of the bacteria in the leaves and flowers originate from the soil in open environments, and the soil is the only traceable source under controlled conditions (Massoni et al., 2021). In this experiment, we took the soybean soil and plant samples at the flowering stage, and our sampling site was located in a warm temperate semi-humid monsoon climate zone with abundant rainwater. The rhizosphere soil and leaf were exposed to the same environmental source. However, at the same time, the phyllosphere, including the leaf surface and leaf endophytic microorganisms, some of which were transferred from the root to the aboveground leaf through the plant stem, is also exposed to environmental sources such as air, dust, and rainwater. Together these factors lead to the fact that the majority of leaf microorganisms originate from rhizosphere microorganisms (Hacquard et al., 2015; Tkacz et al., 2020; Xiong et al., 2021a,b).

### Cropping Systems Sensitive Microbes

Indicator species which are sensitive to specific environmental attributes, are used to monitor environmental changes and assess the efficacy of management (Siddig et al., 2016). Indicator species can show the heterogeneity of group abundance in specific habitats, and can well represent the groups with key regulatory and connection roles in a specific cropping system (De Caceres et al., 2010; Zhang et al., 2021). Indicator species sensitive to crop rotation exist with more soil-plant continuum compartments, indicating that crop rotation may have the potential to change some soil properties such as organic carbon content by changing microbial community composition, and further may be conducive to agricultural activities and improve agricultural productivity (Zhang et al., 2019; Bu et al., 2020). The functional analysis results of indicator species sensitive to crop rotation and intercropping were also interesting, among the microorganisms sensitive to crop rotation, the abundances of plastic degradation and arsenate detoxification microorganisms were relatively high, indicating that they may affect the soil health of cultivated land. The bacterial genera involved in nitrogen cycling were one of the most abundant groups in the intercropping system, so it is necessary to assume that environmental factors affecting these genera also affect nitrogen cycling (Treonis et al., 2010; Legrand et al., 2018). Of course, with regard to the specific functions of these sensitive microorganisms on soil or plants, it is more accurate to test how they affect the soil-plant performance by isolating strains (de Vries et al., 2020).

### Cropping System Effects on Bacteria Co-occurrence

Microorganisms do not grow in isolation, but form complex association networks. Such networks are of great significance for understanding microbial community structure and interactions between members (de Vries et al., 2018; Duran et al., 2018; Ramirez et al., 2018). Our study highlighted how cropping affected the network structure of different compartments of soil-plant continuum, revealing that the complex network of soil related rootzone and rhizosphere compartment were greatly affected by crop rotation, while the plant related endosphere and phyllosphere compartment were affected by continuous cropping. Previous studies have shown that crop rotation has the greatest effect on indicator species of rhizosphere microorganisms, and rhizosphere microorganisms can positively regulate a variety of microorganisms and contribute to crop yield (Sun et al., 2018; Kraut-Cohen et al., 2020; Fan et al., 2021; Wu et al., 2021). Natural connectivity analysis showed that crop rotation increased the competition between rhizosphere microorganisms, thereby enhancing the stability of the network (Foster and Bell, 2012; Coyte et al., 2015; Shi et al., 2020).

Compared with intercropping and continuous cropping, the increased frequency of interaction between *Proteobacteria, Actinomycetes* and other strains under crop rotation indicated the high frequency of interaction between them. Previous studies also found crop rotation could change soil nutrient use efficiency, and that rhizosphere and plant yield were related to productivity (de Vries et al., 2020). In summary, under the rotation system, the soybean rhizosphere had more complex microbial interactions, or more niche overlaps, than other compartments (Berry and Widder, 2014; Zhang et al., 2018). In the future studies, there should be a focus on crop rotation and rhizosphere two factors on crop growth regulation mechanism and the final economic benefits.

### Community Assembly Under Cropping Systems

In the neutral community model (NCM), the dominance test showed that the models had high R^2^ values, and more than 82% of species had frequencies within the predicted ranges. For example, out of all compartments, rhizosphere soil had the fewest ASVs that occurred outside the prediction range, suggesting that the neutral model could better describe the frequency of bacteria in each compartment under different cropping methods. However, we should also consider some non-neutral processes. Less than 18% of bacteria in each compartment were affected by cropping, and their distribution was inconsistent with the neutral prediction (Li P. et al., 2020).

Based on the neutral theory model, we observed that the fitness value (R^2^) and mitigation rate (m) values of NCM in rhizosphere and endosphere were lower than those in other compartments, consistent with previous studies (Burns et al., 2016; Chen et al., 2019). Bacterial community assembly in the rootzone, rhizosphere, endosphere, and phyllosphere compartments under intercropping conditions was mainly affected by stochastic processes, which indicated that intercropping might increase some stochastic processes such as ecological drift and diversification. Combined with the results of NST, the bacterial community assembly in the rhizosphere compartment under intercropping and crop rotation was mainly affected by deterministic processes, while the bacterial community in the continuous cropping system was mainly affected by stochastic processes, which indicated that intercropping and crop rotation might drive environmental selection processes that expose rhizosphere bacteria to larger environmental filters (Vellend et al., 2014; Zhou and Ning, 2017; Li P. et al., 2020). In addition, compared with continuous cropping, crop rotation increased the deterministic processes of bacterial communities in the rootzone and endosphere, indicating that crop rotation might increase environmental heterogeneity, making bacteria more restricted by diffusion and reduce their niche breadth (Pandit et al., 2009; Vellend et al., 2014; Li et al., 2019). Increasing competition and interaction between microorganisms for resources might screen out microbial communities that were more conducive to plant growth and development (Umana et al., 2015; Chen et al., 2021).

## Conclusions

In the present study, our results revealed that the effects of different cropping systems on various microbial properties (α-diversity, community structure, determinism/stochastic model, and interaction network) in different compartment niches were significantly different, and that the rhizosphere showed the greatest impact. Among them, indicator species sensitive to intercropping and crop rotation participated in nitrogen/phosphorus cycle and degradation process, respectively, which has the potential to help plant growth and improve farmland soil fertility. Moreover, crop rotation intensified the competition among root microorganisms, increased deterministic processes of the rhizosphere microbial community, and changed the heterogeneity of the rhizosphere environment. Thus, it affects plant microbial communities and, ultimately, increases the potential to change crop yields and biomass, and overall improves agricultural economic benefits. In order to achieve this goal, in our future work, we will consider screening effective strains or strains combinations that can play a key role in field conditions, and use synthetic microbial communities to analyze how plants regulate their related microbial groups from the aspects of function and mechanism, and how microbial groups feedback to the host. Finally, the complex mechanism of crop-microorganism interaction was revealed to provide insights for the improvement of sustainable agricultural productivity and future crop management.

## Data Availability Statement

The data presented in the study are deposited in the Genome Sequence Archive (Genomics, Proteomics & Bioinformatics 2017) in BIG Data Center (Nucleic Acids Research 2018), Beijing Institute of Genomics (BIG), Chinese Academy of Sciences repository, accession number PRJCA008873, and the database website is http://bigd.big.ac.cn/gsa.

## Author Contributions

SJ developed the original framework. SC performed the experiments with the help of LW, JG, YW, YZ, JQ, ZP, BC, HP, ZW, and HG. Carried out the data analysis and wrote the paper with the help of SJ and GW. All authors contributed intellectual input and assistance to this study and the manuscript preparation.

## Funding

This work was supported by the National Science Foundation of China (Grant No. 41830755), the National Key Research and Development Program of China (Grant No. 2021YFD1900500), and the National Science Foundation for Excellent Young Scholars of China (Grant No. 42122050).

## Conflict of Interest

The authors declare that the research was conducted in the absence of any commercial or financial relationships that could be construed as a potential conflict of interest.

## Publisher's Note

All claims expressed in this article are solely those of the authors and do not necessarily represent those of their affiliated organizations, or those of the publisher, the editors and the reviewers. Any product that may be evaluated in this article, or claim that may be made by its manufacturer, is not guaranteed or endorsed by the publisher.
