# Supplementary material for: Agricultural Management Drive Bacterial Community Assembly in Different Compartments of Soybean Soil-Plant Continuum
Source: Front Microbiol. 2022 May 4;13:868307. doi: 10.3389/fmicb.2022.868307 (PMC9114711; doi:10.3389/fmicb.2022.868307)
Supplement: Supplementary file 1 [file Data_Sheet_1.docx]

***Supplementary Materials***


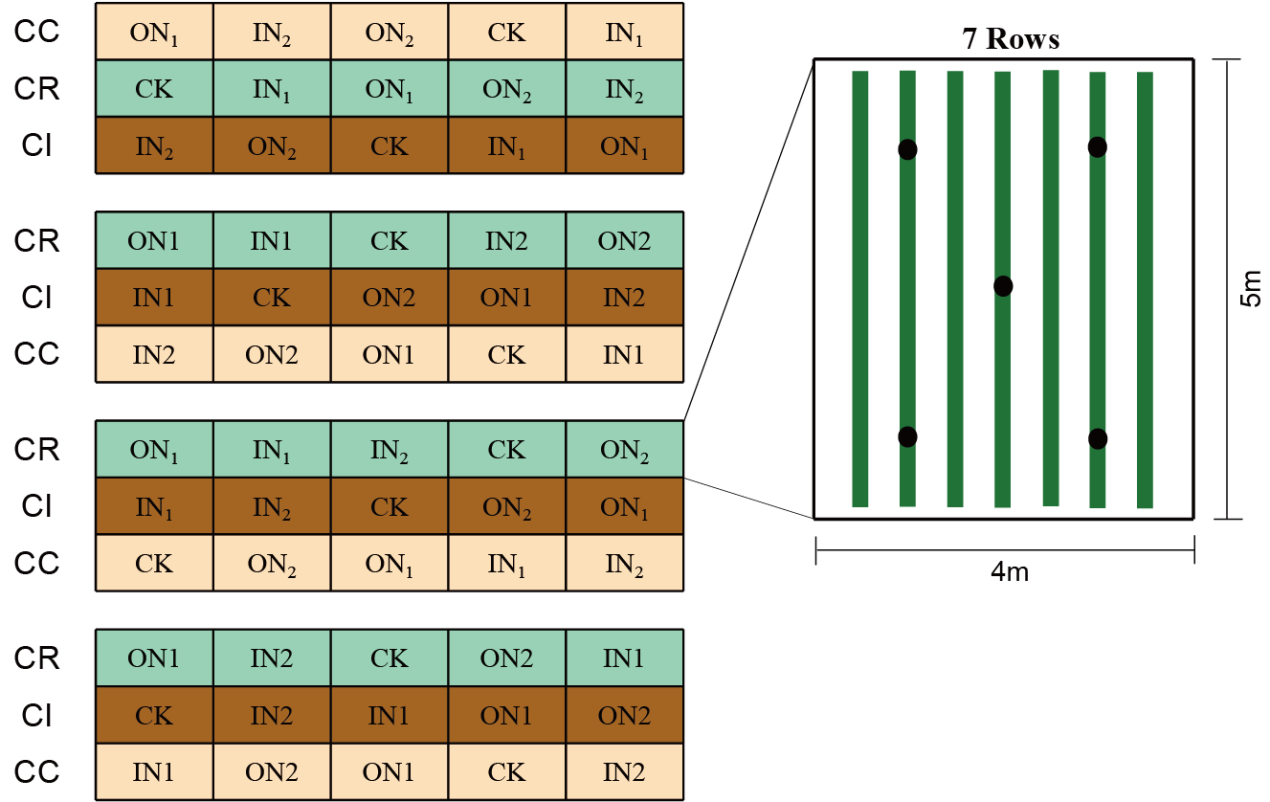


Figure S1. Experimental design and sampling point layout of cropping and fertilization treatment. Three cropping systems included CC: soybean continuous cropping; CR: soybean-wheat rotation cropping; CI: soybean-maize intercropping; five nitrogen fertilizer treatments included CK: plots with no nitrogen fertilizer; IN_1_: 50% inorganic nitrogen fertilizer; IN_2_: 100% inorganic nitrogen fertilizer; ON_1_: 50% organic nitrogen fertilizer, ON_2_: 100% organic nitrogen fertilizer.


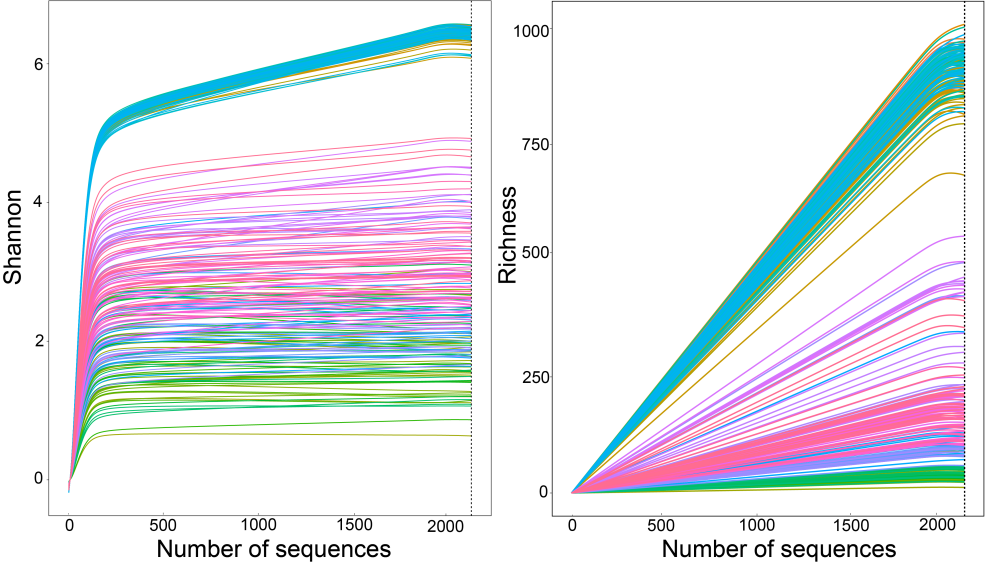


Figure S2. The rarefaction curve of bacterial community in agricultural cropping systems (n=300).


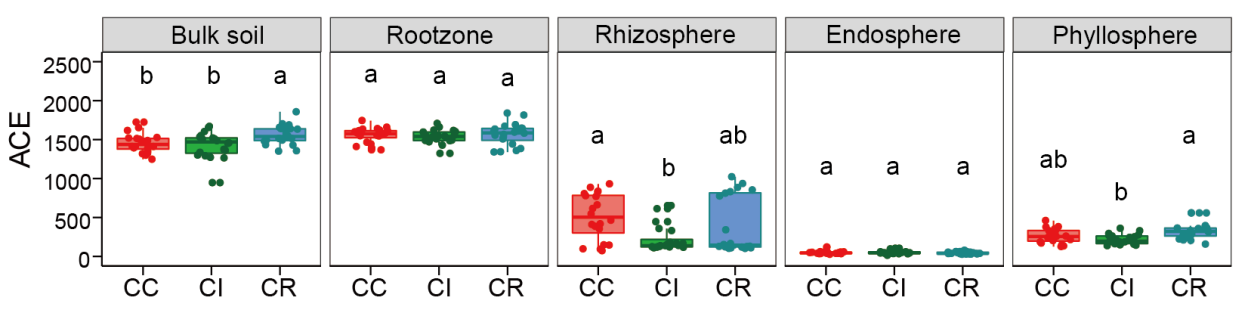


Figure S3. Alpha diversity ACE index of various soil-plant continuum compartments bacteria communities under continuous cropping (CC), intercropping (CI) and rotation (CR) cropping systems. Same lowercase letter indicates no statistically significant (P < 0.05) difference between cropping systems. Significant results were obtained by Wilcoxon test.


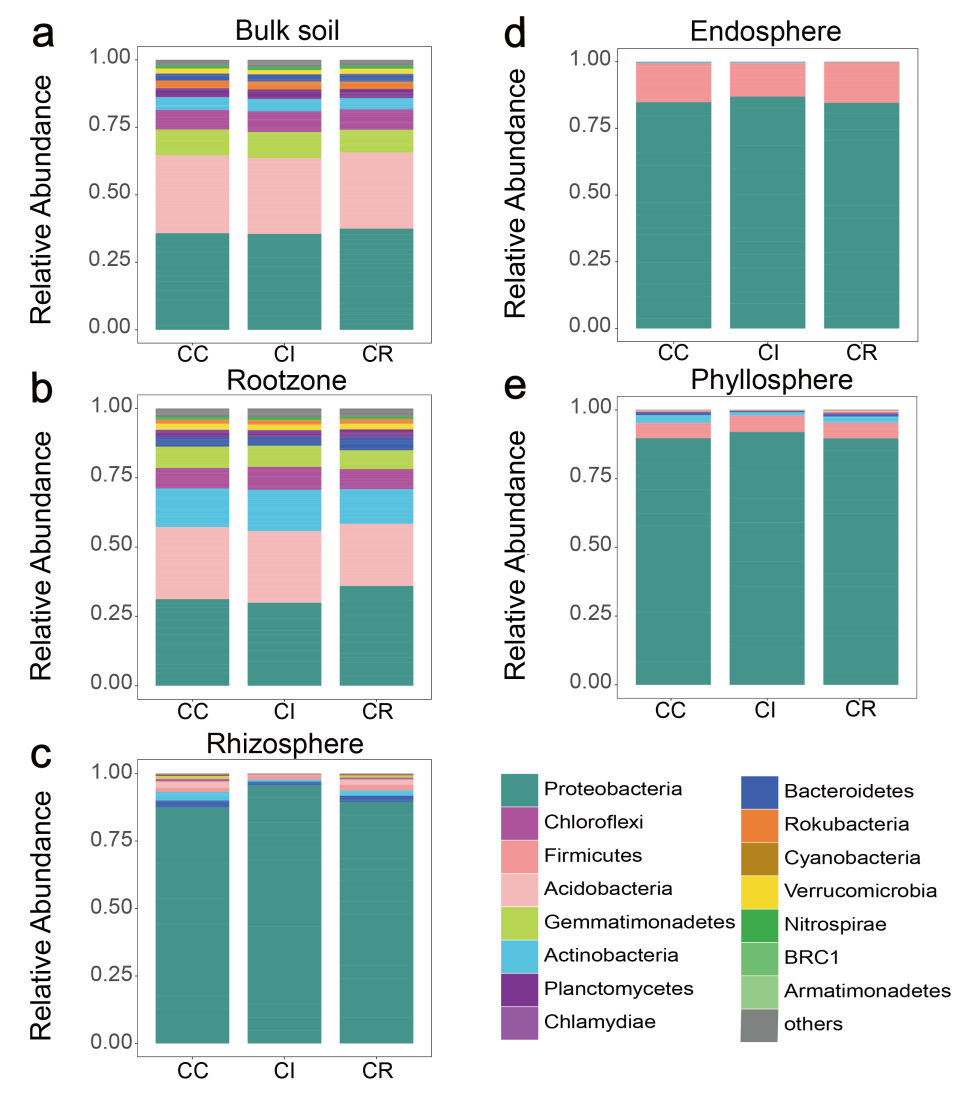


Figure S4. Stack column graph of the top 10 phylums with the highest horizontal relative abundance of bacterial community phylums in soybean soil-plant continuum compartments under three cropping systems.


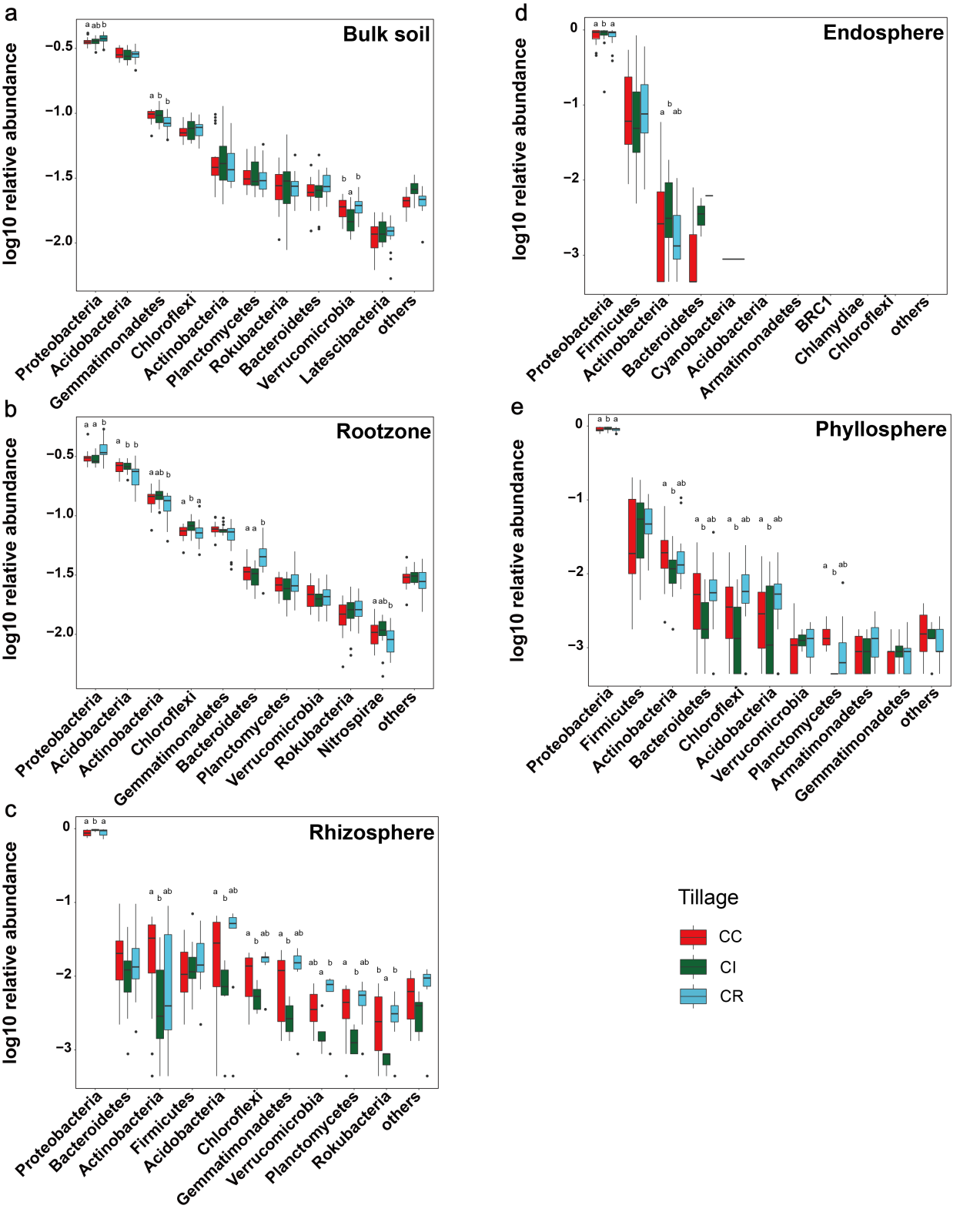


Figure S5. The box plot showed the microorganisms with the relative abundance of top10 at the bacterial community phylum level in each compartment of soybean soil-plant continuum under three cropping systems. Significant results were obtained by Wilcoxon test.


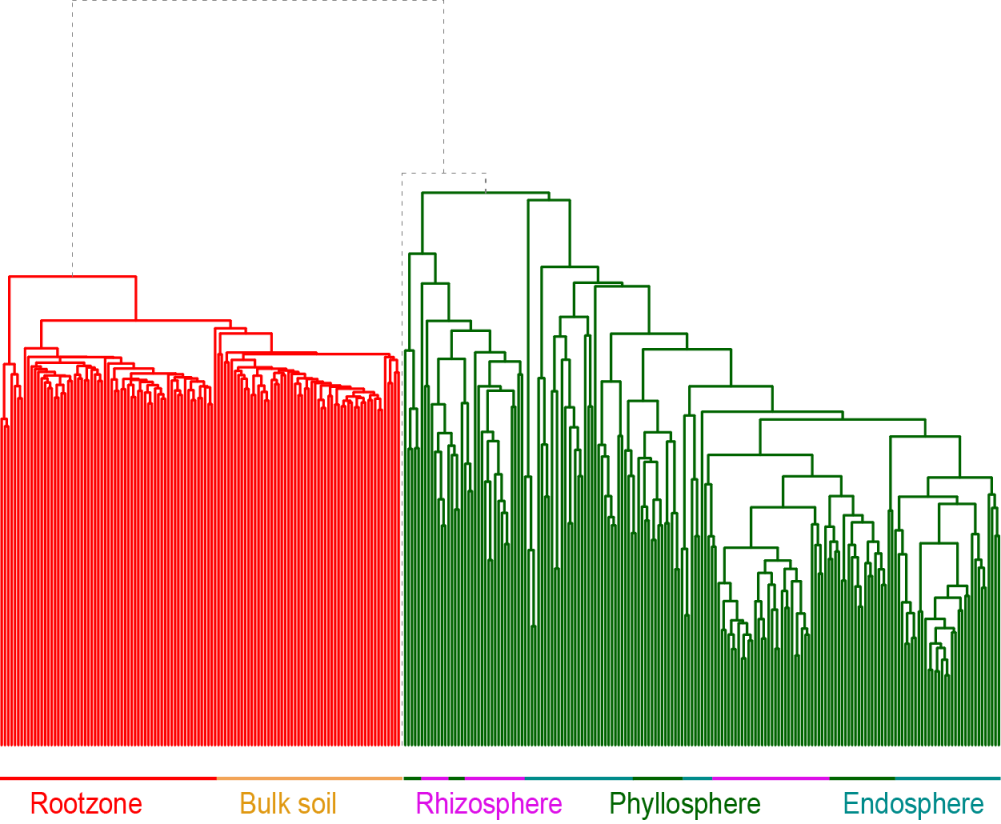


Figure S6. Hierarchical clustering was performed based on the Bray-Curtis distance of all sample bacteria ASVs (n = 300).


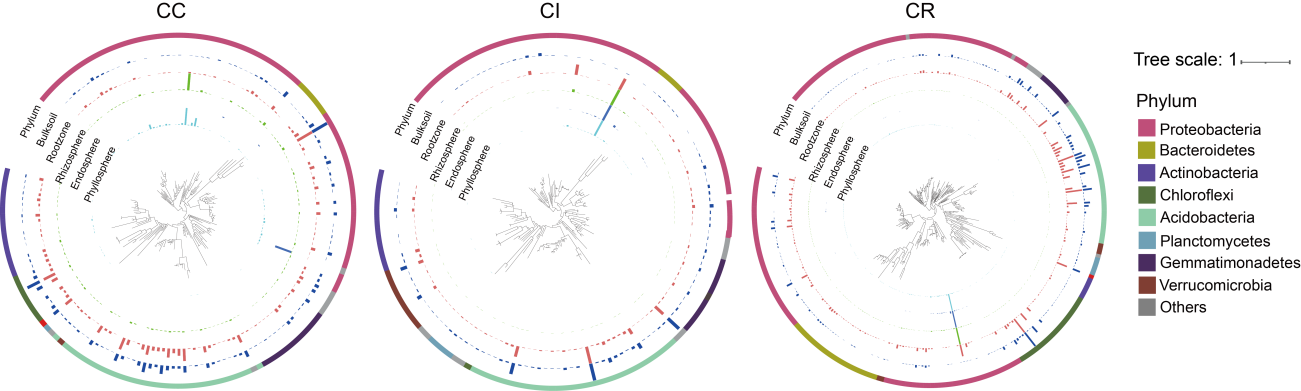


Figure S7. Phylogenetic tree, taxonomic composition and relative abundance of ASvs sensitive to continuous cropping (CC), intercropping (CI) and rotation (CR) systems: 371,171,514 indicator species ASVs sensitived to CC, CI and CR in itols constructed a bacterial phylogenetic tree and marked the relative abundance of each ASV in different parts. Different colors represent phylum level microbial attribution information.


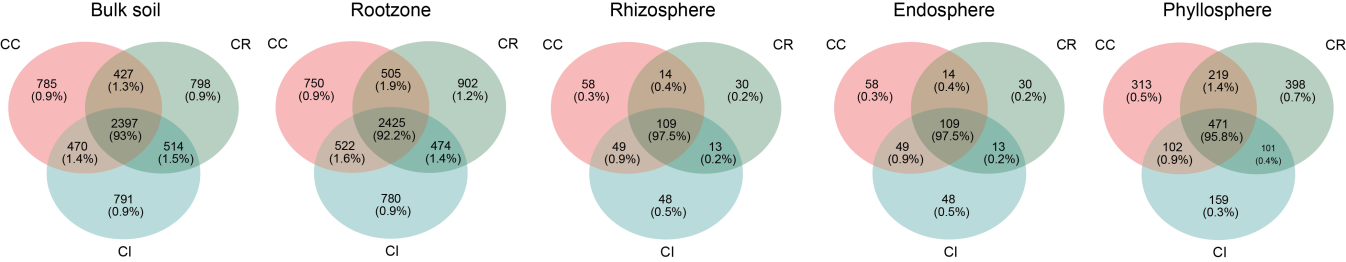


Figure S8. Venn diagram showing the numbers and proportion of different compartments niche shared and specific bacterial ASVs under three cropping systemss.


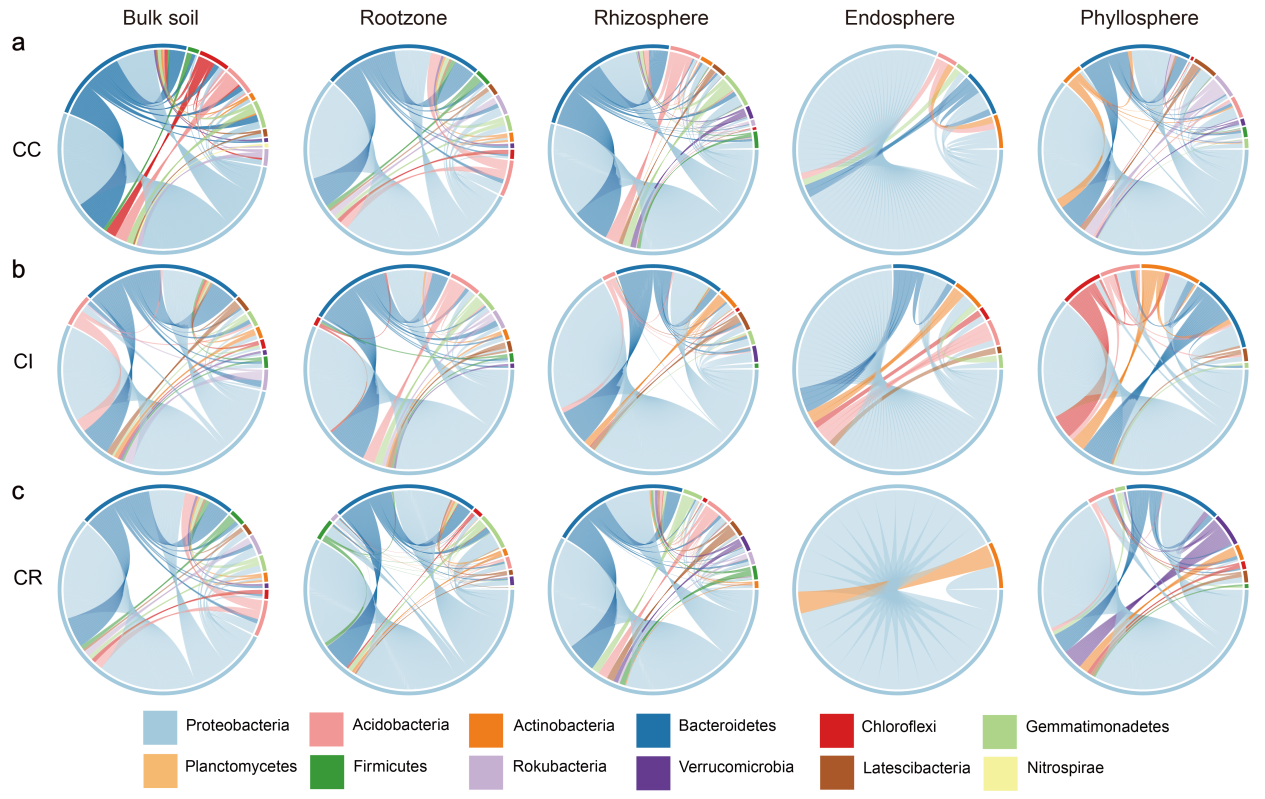


Figure S9. CIRCOS plots showing the distribution of links among the interacting phyla microbe in various compartments under three cropping systemss. Ribbons connecting two segments represent network links between operational taxonomic units assigned to the two segments. The thickness of each ribbon is proportional to the number of links.


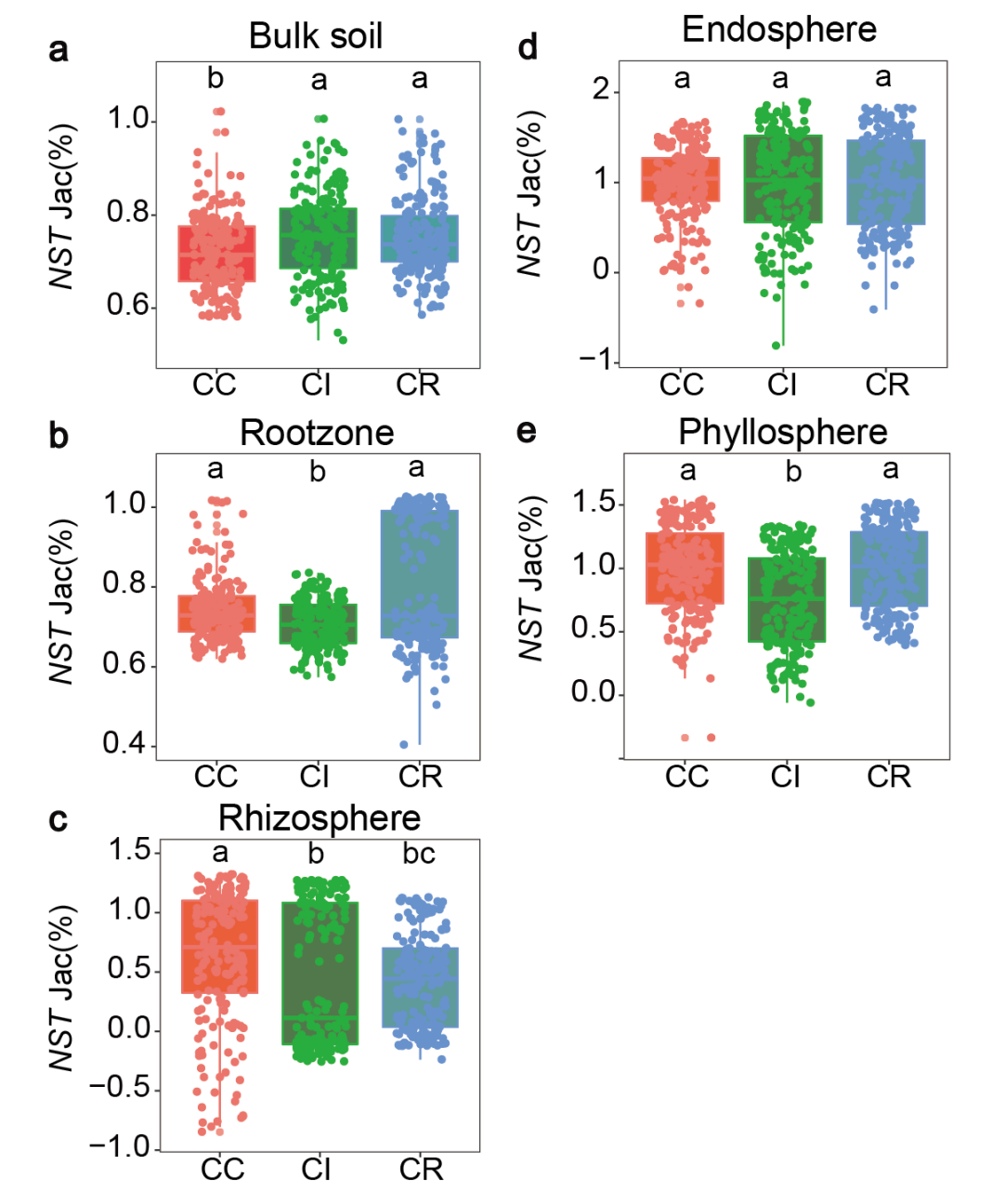


Figure S10. Normalized stochasticity ratio (NST) measurement of community assembly process in soil-plant continuum compartments under three cropping systemss: The normalized stochasticity test (NST) was developed based on Jaccard distance (NSTjac) with 50% as the boundary point between more deterministic (<50%) and more stochastic (>50%) assembly.

Table S1 Effects of cropping systems (CC, CI, CR), fertilize (CK, IN_1_, IN_2_, ON_1_, ON_2_), and niche (Bulk soil, Rootzone, Rhizosphere, Endosphere, Phyllosphere) on the bacterial community based on PERMANOVA.

|  | R^2^ | P.value |
| --- | --- | --- |
| Cropping Systems | 0.00686 | 0.372 |
| Fertilize | 0.00453 | 1 |
| Niche | 0.58249 | 0.001*** |

Table S2 Effects of cropping systems, fertilize on the bacterial community based on PERMANOVA.

|  | Fertilize | | Cropping Systems | |
| --- | --- | --- | --- | --- |
|  | R^2^ | P.value | R^2^ | P.value |
| Bulk soil | 0.06545 | 0.838 | 0.04054 | 0.003** |
| Rootzone | 0.06535 | 0.701 | 0.04949 | 0.003*** |
| Rhizosphere | 0.02730 | 0.999 | 0.07234 | 0.048 * |
| Endosphere | 0.05841 | 0.695 | 0.03498 | 0.413 |
| Phllosphere | 0.05889 | 0.666 | 0.08039 | 0.004** |

Table S3 Diversity of bacterial microbial communities in different soil-plant continuum compartments under different cropping systems. Values are means (SD). Values at the same rows followed by different letters differed significantly at P < 0.05 (Wilcoxon test).

|  |  | CC | CI | CR |
| --- | --- | --- | --- | --- |
| Bulk soil | Richness | 891.20(42.1)b | 885.40(64.7)b | 919.05(43.9)a |
|  | ACE | 1450.80(122.1)b | 1427.92(167.1)b | 1558.15(125.2)a |
| Rootzone | Richness | 925.85(34.5)a | 922.30(29.5)a | 923(44.8)a |
|  | ACE | 1556.51(97.3)a | 1536.47(85.4)a | 1567.11(141.8)a |
| Rhizosphere | Richness | 282.70(151.1)a | 138.40(70.5)b | 230.00(177.7)ab |
|  | ACE | 510.77(294.8)a | 220.89(167.4)b | 396.30(364.6)ab |
| Endosphere | Richness | 43.55(16.6)a | 45.05(17.9)a | 37.70(9.9)a |
|  | ACE | 46.57(18.9)a | 48.95(20.2)a | 42.54(14.3)a |
| Phyllosphere | Richness | 188.20(61.0)ab | 146.40(30.7)b | 205.90(73.7)a |
|  | ACE | 263.45(91.1)ab | 215.86(64.8)b | 322.02(103.7)a |

Table S4 Wilcoxon test of bacterial microbial community diversity in different soil-plant continuum compartments under two cropping systems.

|  |  | CC&CI | CC&CR | CI&CR |
| --- | --- | --- | --- | --- |
| Bulk soil | Richness | 0.797 | 0.021* | 0.012* |
|  | ACE | 0.779 | 0.019* | 0.007** |
| Rootzone | Richness | 0.55 | 0.24 | 0.69 |
|  | ACE | 0.51 | 0.22 | 0.74 |
| Rhizosphere | Richness | 0.009** | 0.351 | 0.675 |
|  | ACE | 0.008** | 0.358 | 0.698 |
| Endosphere | Richness | 0.81 | 0.26 | 0.14 |
|  | ACE | 0.95 | 0.25 | 0.18 |
| Phyllosphere | Richness | 0.06 | 0.185 | 0.0008*** |
|  | ACE | 0.059 | 0.173 | 0.0004*** |

Table S5 Wilcoxon tested the microorganisms with high relative abundance of top10 in different soil-plant continuum compartments under two cropping systems.

|  | Bulk soil | | | Rootzone | | | Rhizosphere | | |
| --- | --- | --- | --- | --- | --- | --- | --- | --- | --- |
| Phylum | CC&CI | CC&CR | CI&CR | CC&CI | CC&CR | CI&CR | CC&CI | CC&CR | CI&CR |
| Proteobacteria | 0.766 | 0.041* | 0.070 | 0.261 | 0.002** | 0.003** | 0.0005*** | 0.379 | 0.0214* |
| Acidobacteria | 0.410 | 0.750 | 0.700 | 0.967 | 0.02* | 0.007** | 0.0006*** | 0.201 | 0.135 |
| Actinobacteria | 0.950 | 0.640 | 0.740 | 0.379 | 0.185 | 0.021* | 0.001** | 0.122 | 0.290 |
| Firmicutes | 0.840 | 0.730 | 1.000 | 0.830 | 0.440 | 0.530 | 0.620 | 0.170 | 0.190 |
| Gemmatimonadetes | 0.870 | 0.001** | 0.01* | 0.516 | 0.081 | 0.208 | 0.0006*** | 0.121 | 0.148 |
| Chloroflexi | 0.530 | 0.210 | 0.970 | 0.008** | 0.490 | 0.009** | 0.0007*** | 0.185 | 0.134 |
| Bacteroidetes | 1.000 | 0.070 | 0.110 | 0.507 | 0.001** | 0.0001*** | 0.056 | 0.482 | 0.317 |
| Planctomycetes | 0.870 | 0.550 | 0.390 | 0.320 | 0.810 | 0.230 | 0.001** | 0.252 | 0.082 |
| Rokubacteria | 0.840 | 0.740 | 0.430 | 0.390 | 0.150 | 0.810 | 0.0002*** | 0.122 | 0.0424* |
| Verrucomicrobia | 0.008** | 0.616 | 0.007** | 0.270 | 0.760 | 0.590 | 0.0004*** | 0.338 | 0.143 |
| Nitrospirae | 0.0005*** | 0.870 | 0.0001*** | 0.303 | 0.151 | 0.034* | 0.0009*** | 0.135 | 0.133 |
| Cyanobacteria | 0.460 | 0.830 | 0.360 | 0.077 | 0.445 | .022* | 0.340 | 0.570 | 0.160 |
| Armatimonadetes | 0.720 | 0.330 | 0.330 | 0.271 | .048* | 0.200 | 0.008* | 0.234 | 0.310 |
| Latescibacteria | 0.320 | 0.490 | 1.000 | 0.088 | 0.796 | 0.058 | 0.002* | 0.223 | 0.143 |
|  | Endosphere | | | Phyllosphere | | |  |  |  |
|  | CC&CI | CC&CR | CI&CR | CC&CI | CC&CR | CI&CR |  |  |  |
| Proteobacteria | 0.660 | 0.860 | 0.400 | 0.240 | 0.800 | 0.120 |  |  |  |
| Acidobacteria | / | / | / | 0.324 | 0.026* | 0.004** |  |  |  |
| Actinobacteria | 0.260 | 0.470 | 0.440 | 0.013* | 0.218 | 0.123 |  |  |  |
| Firmicutes | 0.580 | 0.750 | 0.350 | 0.560 | 0.180 | 0.780 |  |  |  |
| Gemmatimonadetes | / | / | / | 0.767 | 0.028* | 0.016* |  |  |  |
| Chloroflexi | / | / | / | 0.052 | 0.315 | 0.005** |  |  |  |
| Bacteroidetes | 0.970 | 0.320 | 0.350 | 0.015* | 0.674 | 0.002** |  |  |  |
| Planctomycetes | / | / | / | 0.007** | 0.146 | 0.195 |  |  |  |
| Rokubacteria | / | / | / | / | / | / |  |  |  |
| Verrucomicrobia | / | / | / | 0.042* | 0.167 | 0.001** |  |  |  |
| Nitrospirae | / | / | / | 0.983 | 0.071 | 0.059 |  |  |  |
| Cyanobacteria | 0.340 | 0.340 | / | 0.081 | 0.301 | 0.008** |  |  |  |
| Armatimonadetes | / | / | / | 0.030 | 0.058 | 0.015 |  |  |  |
| Latescibacteria | / | / | / | / | / | / |  |  |  |

Table S6 Wilcoxon test of beta diversity under cropping systems measures in different soil-plant continuum compartments.

|  | Treatment | F.Model | R^2^ | P.Value |
| --- | --- | --- | --- | --- |
| Bulk soil | CC&CR | 1.27 | 0.03 | 0.01* |
|  | CC&CI | 1.28 | 0.03 | 0.01* |
|  | CI&CR | 1.06 | 0.03 | 0.13 |
| Rootzone | CC&CR | 1.42 | 0.04 | 0.04* |
|  | CC&CI | 1.83 | 0.05 | 0.01* |
|  | CI&CR | 1.18 | 0.03 | 0.02* |
| Rhizosphere | CC&CR | 2.20 | 0.05 | 0.09 |
|  | CC&CI | 1.38 | 0.04 | 0.25 |
|  | CI&CR | 3.35 | 0.08 | 0.04* |
| Endosphere | CC&CR | 0.65 | 0.02 | 0.68 |
|  | CC&CI | 0.53 | 0.01 | 0.85 |
|  | CI&CR | 0.34 | 0.01 | 0.99 |
| Phyllosphere | CC&CR | 2.73 | 0.07 | 0.04* |
|  | CC&CI | 1.63 | 0.04 | 0.12 |
|  | CI&CR | 3.47 | 0.08 | 0.01* |

|  | Bulk soil | | | Rootzone | | | Rhizosphere | | | Endosphere | | | Phyllosphere | | |
| --- | --- | --- | --- | --- | --- | --- | --- | --- | --- | --- | --- | --- | --- | --- | --- |
| Phylum | CC | CI | CR | CC | CI | CR | CC | CI | CR | CC | CI | CR | CC | CI | CR |
| Proteobacteria | 16 | 21 | 49 | 25 | 16 | 73 | 36 | 9 | 16 | 3 | 3 | 2 | 22 | 13 | 29 |
| Actinobacteria | 1 | 1 | 2 | 5 | 12 | 3 | 14 | / | / | / | / | / | 2 | 1 | 1 |
| Acidobacteria | 13 | 14 | 17 | 22 | 17 | 8 | 22 | / | 16 | / | / | / | / | / | 1 |
| Chloroflexi | 1 | 3 | 9 | 2 | 6 | 10 | 6 | / | 4 | / | / | / | 1 | / | 4 |
| Bacteroidetes | 1 | 2 | 6 | 3 | 2 | 19 | 3 | 1 | 1 | / | / | / | 2 | / | 3 |
| Planctomycetes | 1 | 3 | 1 | 2 | 1 | 4 | / | / | / | / | / | / | / | / | / |
| Gemmatimonadetes | 8 | 4 | 2 | 14 | 6 | 6 | 5 | / | 3 | / | / | / | / | / | / |
| Firmicutes | / | / | / | 2 | 1 | / | / | / | 1 | / | / | / | 1 | / | / |
| Armatimonadetes | / | / | / | 2 | 1 | / | / | / | / | / | / | / | / | / | / |
| Rokubacteria | 1 | 3 | 2 | / | 1 | 1 | 3 | / | 3 | / | / | / | / | / | / |
| Latescibacteria | / | 1 | 1 | 1 | 1 | / | 1 | / | / | / | / | / | / | / | / |
| Verrucomicrobia | / | / | 3 | 2 | 1 | 1 | 1 | / | 1 | / | / | / | / | / | / |
| sum | 42 | 54 | 92 | 83 | 67 | 125 | 92 | 10 | 46 | 3 | 3 | 2 | 28 | 14 | 39 |

Table S7 Quantitative Distribution of phylum level microorganisms sensitive to cropping systems in different compartments.

|  |  | CC | | CI | | CR | |
| --- | --- | --- | --- | --- | --- | --- | --- |
|  | Total sequence | sequence | percent | sequence | percent | sequence | percent |
| Bulk soil | 135600 | 1854 | 1.37 | 3013 | 2.22 | 4640 | 3.42 |
| Rootzone | 135600 | 8178 | 6.03 | 6391 | 4.71 | 7823 | 5.77 |
| Rhizosphere | 135600 | 3099 | 2.29 | 9673 | 7.13 | 4620 | 3.41 |
| Endosphere | 135600 | 106 | 0.08 | 1548 | 1.14 | 82 | 0.06 |
| Phyllosphere | 135600 | 3755 | 2.77 | 51131 | 37.71 | 50048 | 36.91 |
| sum | 678000 | 16992 | 2.51 | 71756 | 10.58 | 67213 | 9.91 |

Table S8 Proportion of ASVs in total sequence sensitive to cropping systemss in various compartments (effect size).

Table S9 Number of ASVs sensitive to cropping systemss in different compartments that are specific and shared to complete dataset.

|  | CC | | CI | | CR | |
| --- | --- | --- | --- | --- | --- | --- |
|  | specific | shared | specific | shared | specific | shared |
| Bulk soil | 8 | 17 | 5 | 28 | 9 | 50 |
| Rootzone | 5 | 24 | 4 | 31 | 21 | 52 |
| Rhizosphere | 7 | 37 | 1 | 4 | 2 | 6 |
| Endosphere | 1 | 1 | 1 | 1 | 0 | 2 |
| Phyllosphere | 3 | 16 | 2 | 7 | 4 | 15 |

Table S10 Results of the Kolmogorov-Smirnov test comparing bootstrapped node attributes of bacteria networks under three cropping practice.

|  | Treatment | degree | betweenness | closeness |
| --- | --- | --- | --- | --- |
| Bulk soil | CC&CI | 1.07*** | 26.88*** | 6.28*** |
|  | CC&CR | 1.07*** | 5.99*** | 2.47*** |
|  | CI&CR | 1.00* | 0.22*** | 0.39*** |
| Rootzone | CC&CI | 1.01 | 1.66*** | 1.88*** |
|  | CC&CR | 0.18*** | 0.08*** | 0.16*** |
|  | CI&CR | 0.18*** | 0.05*** | 0.09*** |
| Rhizosphere | CC&CI | 1.61*** | 2.27*** | 0.78*** |
|  | CC&CR | 0.96*** | 1.12*** | 1.00*** |
|  | CI&CR | 0.601*** | 0.49*** | 1.29*** |
| Endosphere | CC&CI | 1.18*** | 2.36*** | 2.25*** |
|  | CC&CR | 1.60*** | 3.87*** | 1.43*** |
|  | CI&CR | 1.36*** | 1.64** | 0.64*** |
| Phyllosphere | CC&CI | 1.70*** | 1.86*** | 1.07*** |
|  | CC&CR | 1.93*** | 0.57*** | 1.03*** |
|  | CI&CR | 1.14*** | 0.31*** | 0.96*** |

Table S11 Main topological properties of microbial co-occurrence network in different compartments under three cropping systems. “BSCC, BSCI, BSCR” represents the network topology parameters of Bulk soil compartments under continuous cropping, intercropping and crop rotation respectively, and other abbreviated analogy.

|  | BSCC | BSCI | BSCR | RZCC | RZCI | RZCR | RSCC | RSCI | RSCR |
| --- | --- | --- | --- | --- | --- | --- | --- | --- | --- |
| num.edges | 172 | 139 | 140 | 153 | 149 | 1140 | 1000 | 298 | 835 |
| num.pos.edges | 103 | 78 | 82 | 99 | 104 | 805 | 776 | 193 | 734 |
| num.neg.edges | 69 | 61 | 58 | 54 | 45 | 335 | 224 | 105 | 101 |
| num.vertices | 236 | 204 | 205 | 216 | 213 | 292 | 140 | 67 | 112 |
| connectance | 0.01 | 0.01 | 0.01 | 0.01 | 0.01 | 0.03 | 0.10 | 0.13 | 0.13 |
| average.degree | 1.46 | 1.36 | 1.37 | 1.42 | 1.40 | 7.81 | 14.29 | 8.90 | 14.91 |
| average.path.length | 3.94 | 1.62 | 2.06 | 2.34 | 1.92 | 4.02 | 3.41 | 2.71 | 3.28 |
| edge.connectivity | 0.08 | 0.30 | 0.20 | 0.21 | 0.26 | 0.72 | 0.66 | 0.56 | 0.74 |
| clustering.coefficient | 70 | 74 | 71 | 72 | 73 | 47 | 2 | 3 | 3 |
| centralization.degree | 0.02 | 0.02 | 0.01 | 0.02 | 0.02 | 0.14 | 0.18 | 0.32 | 0.27 |
| centralization.betweenness | 0.011 | 0.000 | 0.002 | 0.003 | 0.002 | 0.032 | 0.194 | 0.086 | 0.173 |
| centralization.closeness | 0.001 | 0.000 | 0.000 | 0.001 | 0.000 | 0.004 | 0.050 | 0.064 | 0.050 |
|  | ESCC | ESCI | ESCR | PSCC | PSCI | PSCR |  |  |  |
| num.edges | 48 | 49 | 14 | 379 | 128 | 164 |  |  |  |
| num.pos.edges | 47 | 49 | 14 | 335 | 97 | 144 |  |  |  |
| num.neg.edges | 1 | 0 | 0 | 44 | 31 | 20 |  |  |  |
| num.vertices | 45 | 54 | 21 | 110 | 63 | 92 |  |  |  |
| connectance | 0.05 | 0.03 | 0.07 | 0.06 | 0.07 | 0.04 |  |  |  |
| average.degree | 2.13 | 1.81 | 1.33 | 6.89 | 4.06 | 3.57 |  |  |  |
| average.path.length | 2.50 | 2.05 | 1.32 | 3.19 | 2.41 | 3.62 |  |  |  |
| edge.connectivity | 0.62 | 0.60 | 0.43 | 0.48 | 0.55 | 0.33 |  |  |  |
| clustering.coefficient | 11 | 16 | 8 | 10 | 10 | 8 |  |  |  |
| centralization.degree | 0.07 | 0.06 | 0.03 | 0.19 | 0.16 | 0.17 |  |  |  |
| centralization.betweenness | 0.037 | 0.016 | 0.009 | 0.113 | 0.061 | 0.198 |  |  |  |
| centralization.closeness | 0.010 | 0.005 | 0.007 | 0.017 | 0.016 | 0.016 |  |  |  |

Table S12 Number of network connections in different compartments of the microbial phylum level under three cropping systems. “BSCC, BSCI, BSCR” represents the network topology parameters of Bulk soil compartments under continuous cropping, intercropping and crop rotation respectively, and other abbreviated analogy.

|  | BSCC | BSCI | BSCR | RZCC | RZCI | RZCR | RSCC | RSCI | RSCR | ESCC | ESCI | ESCR | PSCC | PSCI | PSCR |
| --- | --- | --- | --- | --- | --- | --- | --- | --- | --- | --- | --- | --- | --- | --- | --- |
| Proteobacteria | 91 | 76 | 81 | 87 | 83 | 797 | 556 | 213 | 505 | 41 | 36 | 13 | 253 | 77 | 111 |
| Acidobacteria | 41 | 35 | 32 | 37 | 38 | 212 | 240 | 54 | 147 | 3 | 5 | / | 60 | 19 | 19 |
| Actinobacteria | 6 | 3 | 4 | 5 | 5 | 36 | 49 | 2 | 36 | 1 | 1 | / | 1 | 1 | 3 |
| Gemmatimonadetes | 10 | 2 | 2 | 2 | 2 | 14 | 3 | 1 | 2 | / | 1 | / | 1 | 14 | 3 |
| Chloroflexi | 10 | 8 | 9 | 9 | 9 | 11 | 79 | 10 | 44 | 2 | 3 | / | 3 | 5 | 3 |
| Bacteroidetes | 3 | 2 | 4 | 3 | 2 | 25 | 15 | / | 25 | / | / | / | 1 | / | 1 |
| Firmicutes | 2 | 3 | 2 | 2 | 2 | 22 | 3 | 10 | 7 | 1 | 2 | 1 | 21 | 10 | 5 |
| Verrucomicrobia | 1 | 1 | 1 | 1 | 1 | 2 | 29 | / | 22 | / | / | / | 5 | / | 16 |
| Latescibacteria | 1 | / | / | / | / | / | / | / | / | / | / | / | / | / | / |
| Rokubacteria | 5 | 5 | 3 | 5 | 4 | 5 | 8 | / | 14 | / | / | / | 24 | / | / |
| Nitrospirae | 2 | 4 | 2 | 2 | 3 | 15 | 18 | 8 | 33 | / | 1 | / | 10 | 2 | 3 |
| sum | 172 | 139 | 140 | 153 | 149 | 1140 | 1000 | 298 | 835 | 48 | 49 | 14 | 379 | 128 | 164 |
